# DeepIMAGER: Deeply Analyzing Gene Regulatory Networks from scRNA-seq Data

**DOI:** 10.3390/biom14070766

**Published:** 2024-06-27

**Authors:** Xiguo Zhou, Jingyi Pan, Liang Chen, Shaoqiang Zhang, Yong Chen

**Affiliations:** 1 College of Computer and Information Engineering, Tianjin Normal University, Tianjin 300387, China; jxzxg@tjnu.edu.cn (X.Z.); 2111090002@stu.tjnu.edu.cn (J.P.); 2210090012@stu.tjnu.edu.cn (L.C.); 2 Department of Biological and Biomedical Sciences, Rowan University, Glassboro, NJ 08028, USA

**Keywords:** scRNA-seq, deep learning, gene regulatory networks, cell types

## Abstract

Understanding the dynamics of gene regulatory networks (GRNs) across diverse cell types poses a challenge yet holds immense value in unraveling the molecular mechanisms governing cellular processes. Current computational methods, which rely solely on expression changes from bulk RNA-seq and/or scRNA-seq data, often result in high rates of false positives and low precision. Here, we introduce an advanced computational tool, DeepIMAGER, for inferring cell-specific GRNs through deep learning and data integration. DeepIMAGER employs a supervised approach that transforms the co-expression patterns of gene pairs into image-like representations and leverages transcription factor (TF) binding information for model training. It is trained using comprehensive datasets that encompass scRNA-seq profiles and ChIP-seq data, capturing TF-gene pair information across various cell types. Comprehensive validations on six cell lines show DeepIMAGER exhibits superior performance in ten popular GRN inference tools and has remarkable robustness against dropout-zero events. DeepIMAGER was applied to scRNA-seq datasets of multiple myeloma (MM) and detected potential GRNs for TFs of *RORC*, *MITF*, and *FOXD2* in MM dendritic cells. This technical innovation, combined with its capability to accurately decode GRNs from scRNA-seq, establishes DeepIMAGER as a valuable tool for unraveling complex regulatory networks in various cell types.

## 1. Introduction

Exploring the spatial and temporal coordination of genes and other biomolecules within cells is foundational research that not only enhances our comprehension of normal biological processes but also provides crucial insights into the underlying mechanisms of various pathological conditions [1,2]. Gene regulatory networks (GRNs) emerge as a pivotal and indispensable tool to describe the relationships among genes [3]. These networks serve as molecular interaction maps within cells, enabling us to understand the transcriptional regulation of gene expression governing the behavior of living organisms [2,4,5]. Thus, it is important to decipher these GRNs and their dynamics to shed light on the machinery responsible for cellular functions, tissue development, and organismal homeostasis. In medical research, elucidating aberrant GRNs associated with various disorders has the potential to revolutionize diagnostics and treatment approaches, benefiting therapeutic interventions targeted at specific regulatory nodes within the network [2,4,6].

Various computational methods have been developed for reconstructing GRNs using both single-cell RNA sequencing (scRNA-seq) and bulk RNA sequencing (RNA-seq) data. Notable approaches include GENIE3 [7], PIDC [8], SCODE [9], PPCOR [10], SINCERITIES [11], SCENIC [12], SCENIC+ [13] and SINGE [14]. For instance, SCODE [9] utilizes differential equations to model the dynamic changes in gene expression levels over time or under different conditions. PIDC [8] employs gene correlation metrics to measure the strength of association between pairs of genes and then applies information theory-based approaches to identify causal relationships among genes. SCENIC [12] employs gene correlation metrics to quantify the co-expression relationships between genes and uses this information, along with transcription factor (TF) binding motif analysis, to infer regulatory interactions and identify key transcriptional regulators driving cell identity and function. Its updated version, SCENIC+ [13], can further integrate scATAC-seq, scRNA-seq, and TF-binding motifs to predict GRNs. SINCERITIES [11] and SINGE [14] utilize correlation ensemble methods in conjunction with pseudo-time ordering of cells. These methods have significantly contributed to our understanding of generic GRNs in experimental settings [15]. However, improving performance in reconstructing cell-specific gene networks is an outstanding research question due to two major limitations [16,17]. First, the accuracy of GRN inference methods is predominantly affected by high rates of false-positive and/or false-negative interactions in many correlation-based methods like SCENIC [12] and SCODE [9]. Second, inferring GRNs from scRNA-seq data presents computational challenges in handling high-dimensional, noisy data and identifying causal relationships between genes. While some methods originally designed for bulk RNA-seq data, such as GENIE3 [7], have been adapted for scRNA-seq data, their performance is often suboptimal [17], frequently affected by the high prevalence of dropout-zero expression values in scRNA-seq data [18,19].

To address these limitations in GRN inference from scRNA-seq data, researchers have explored two promising strategies: the utilization of deep learning-based methods and the integration of multiple omics data types. Deep learning-based methods leverage advanced neural network architectures to capture complex and non-linear dependencies within gene expression data. These methods include DeepSEM [20], CNNC [21], DeepDRIM [22], dynDeepDRIM [23], GENELink [24], GNNLink [25], scTIGER [26], scGeneRAI [18], DeepMCL [27], and STGRNS [28], which can automatically learn intricate patterns and interactions that may be missed by conventional techniques. To further enhance the reliability of GRN inference, researchers have turned to the integration of diverse omics data types. This approach acknowledges that gene regulation is a multi-layered process involving various molecular components. By combining data from different omics levels, such as RNA-seq, ChIP-seq, and proteomics, a more comprehensive view of gene regulation can be obtained. In this direction, CNNC, DeepDRIM, dynDeepDRIM, GENELink, DeepMCL, and STGRNS are all supervised learning methods that use known cell-specific TF-gene interactions to reconstruct GRNs.

While researchers have made significant progress in constructing more accurate and biologically relevant GRNs through the combination of deep learning techniques and integration of multi-omics data types, additional challenges persist in real-world applications [16,17]. First, the selection of an appropriate deep learning architecture or method for a specific GRN inference task can be a non-trivial undertaking. Different datasets and research questions may necessitate distinct models, making it a daunting task to identify the optimal one. Additionally, the complexity of integrating multi-omics data types adds another layer of complexity to the process. It demands careful consideration of data alignment, normalization, and feature selection. Moreover, omics data, including scRNA-seq and ChIP-seq, frequently contain noise and experimental artifacts. Some deep learning models exhibit sensitivity to noisy data, potentially resulting in the extraction of spurious interactions. Specifically, CNNC [21] only considers data expression of target TF-gene pairs as input to predict direct regulatory relationships, which eventually leads to false positives in practical applications. Although DeepDRIM [22] and dynDeepDRIM [23] consider the neighborhood context of the target TF-gene pairs, their core neural networks are based on VGGnet [29], which has no residual structure and may lead to network degradation and output errors [30]. DeepMCL trains a deep Siamese convolutional neural network with a contrastive loss to learn the low-dimensional embedding of each gene pair [27]. Both GENELink and GNNLink use graph attention network models to reconstruct GRNs in a low-dimensional gene space [24,25]. It is worth noting that low-dimensional embeddings may lead to the loss of some co-expression information of gene pairs. Meanwhile, STGRNS is an interpretable transformer-based GRN inference method suitable for static or time-series scRNA-seq datasets, but its prediction accuracy is unstable [28].

In this study, we introduce DeepIMAGER (Deeply Integrating Multi-omics to Analyze Gene Expression Regulations), an advanced deep learning-based method for inferring GRNs from scRNA-seq datasets. DeepIMAGER leverages the concept of translation of gene pairs into images and employs the ResNet50 convolutional neural network [31] for prediction. The model is trained using known TF-gene interaction pairs as positive samples and non-TF-gene pairs as negative samples. It searches potential regulatory relationships from these highly correlated neighbor images, thus mitigating false positives stemming from transitive interactions. Comparison with other methods on six real scRNA-seq datasets reveals that DeepIMAGER achieves the highest prediction accuracy with the lowest loss values on the test sets, outperforming ten popular GRN inference methods. When applied to scRNA-seq datasets of Multiple Myeloma (MM) patients, we identified potential regulations in GRNs of three TFs, *RORC*, *MITF*, and *FOXD2* in dendritic cells, including many known regulations related to MM pathology.

## 2. Materials and Methods

### 2.1. Overview of DeepIMAGER

**Figure 1:**
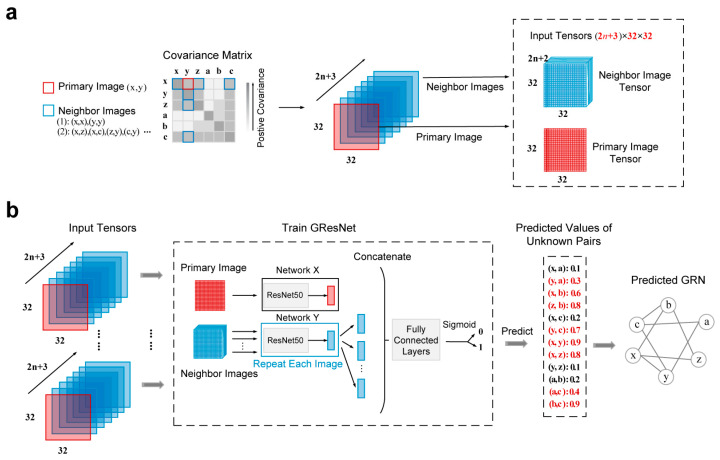
Overview of DeepIMAGER. (**a**) The joint expression (x,y) of genes x and y is represented by a primary image, while the joint expression (x, i) or (y, i), where i is a gene with high covariance with gene x or gene y, is represented by a neighbor image. The total number of a primary image and its neighbor images are 2n+3. (**b**) The workflow of DeepIMAGER encompasses the training model and the predicted GRN. It uses the network X and network Y  to construct the primary image and its neighbor images, respectively, with specific network structures shown in Figure S1.

To efficiently infer cell-specific GRNs from scRNA-seq data, DeepIMAGER translates the expression profiles of gene pairs into images and applies ResNet50 [31] to these images to efficiently predict the potential regulatory relationships among genes. Figure 1 illustrates the DeepIMAGER workflow for predicting interactions associated with a pair of genes, x, and y, corresponding to a target TF-gene pair.

First, for each TF-target gene pair (x, y), the joint expression of (x, y) is transformed into a two-dimensional (2D) histogram, defined as a primary image with 32 × 32 bins (Figure 1a). The intensity of each bin represents the number of co-occurring cells whose gene expression values fall within the bin interval. The joint expression of gene x (or gene y) and one of its top n genes with positive covariance is transformed into a 32 × 32 2D histogram as a neighbor image of the primary image. Including the two self-images transformed from (x, x) and (y, y), there are a total of (2n+2) neighbor images for the primary image. These neighbor images capture critical neighboring information of the primary image, which is essential for distinguishing direct interactions from transitive interactions, effectively reducing errors caused by transitive gene-gene interactions. Second, for each target TF-gene pair (x,y), the tensors of the primary image and its associated (2n+2) neighbor images are separately input to two deep neural networks X and Y, which are slight modifications of ResNet50 (Figure 1b). For each image tensor, the network output is a 512-dimensional embedding vector. Third, the (2n+3) embedding vectors of a primary image and its neighbor images are concatenated into a 512×(2n+3)-dimensional vector. Finally, the concatenated vector undergoes compression through fully connected layers, and binary classification is performed using the sigmoid function. These neural networks are trained using known TF-gene interactions obtained from publicly available cell-specific ChIP-seq data, and the predicted interactions are represented as directed edges with high confidence scores, defined within the range of 0 and 1.

### 2.2. Data Preprocessing and 2D Representation of Gene-Gene Joint Expression

We used SCANPY (version 1.9.6) [32] to preprocess the raw scRNA-seq expression matrix. In particular, we first used the “scanpy.pp.nornalized_total” function to normalize the expression counts per cell by the total counts across all genes with a size factor of $10^{4}$, and then used the “scanpy.pp.log1p” function to perform a log-transformation of the normalized matrix. Subsequently, we used the “scanpy.pp.highly_variable_genes” function to select the top 500 highly variable genes (HVGs). Finally, for each target gene x, the covariance of expression vectors across all cells between x and any other gene was calculated, and top n genes with high positive covariance were selected.

To avoid extreme values, the top 5% of the largest elements for each gene are all reset as the minimum value of these top elements. For each gene, we divided its preprocessed expression values in all cells equally into 32 bins. For two given genes (x,y), according to their bin divisions, we created a 2D histogram H(x,y) consisting of 32×32 bins, where each bin value $H_{ij}$ represents the number of cells where the two genes’ preprocessed expression values fall within the bin. Finally, we performed log-normalization as ${\tilde{H}}_{\mathrm{ij}}=\log_{10}\left(1+10H_{ij}/\sum_{ij}H_{ij}\right)$ for each $H_{ij}$ to avoid extreme values in the constructed histogram.

### 2.3. Network Structure of DeepIMAGER

The network structure of DeepIMAGER consists of two identical parts, namely network X and network Y, designed for processing primary images and neighbor images, respectively. Each of these networks, whether X or Y, comprises 48 convolutional layers, accompanied by 1 max pooling layer (MaxPool), 1 average pooling layer (AvgPool), and a fully connected layer (FC), which are similar architectural elements from ResNet50 [31]. In DeepIMAGER, the outputs from both network X and network Y are concatenated and subsequently compressed by two fully connected layers, each implementing a 50% dropout rate. The framework employed in networks X and Y, features a parent module, four stages, an average pooling layer, and a fully connected layer (Figure S1a).

The parent module, referred to as a stem block (Figure S1b), comprises three 3 × 3 convolutional layers, with a stride of 2 in the first convolution and a max pooling layer for down-sampling. The output features of the stem block are half the size of the input features. Each of the four stages includes two types of Bottleneck structures, denoted as Block1 and Block2. Block1 encompasses two paths that form a down-sampling module: the left path comprises three 3 × 3 convolutional layers for learning new features, while the right path consists of a 1 × 1 convolutional layer and an AvgPool layer, processing the input to match the size and scale of the output from the left path (Figure S1c). For stages 2 to 4, Block1 with a parameter “stride = 2” reduces the feature map size by half. Block2, similar to Block1, consists of two paths; however, in Block2, the right path serves as a short-cut connection, forming the residual module with the left path (Figure S1d). These residual structures in Block1 and Block2 allow input data to traverse the network, ensuring that even small errors can propagate through the network. Moreover, after each convolutional layer, ResNet incorporates the rectified linear activation function (ReLU) (Figure S1c,d), promoting linear isolation between consecutive convolutional layers, ensuring that each convolutional layer can effectively fulfill its role. After passing through four stages but before reaching the final fully connected layer, there is a global average pooling layer with a 50% dropout rate, which serves to mitigate overfitting to some extent during the training process (Figure S1a).

For a primary image and its (2n+2) neighbor images, the outputs of networks *X* and *Y* yield (2n+3) 512-dimensional vectors. These vectors are concatenated, compressed through two fully connected layers, each applying a 50% dropout rate, and classified using a sigmoid function with binary cross-entropy loss. The weights of networks X and Y are initialized randomly and trained using small-batch stochastic gradient descent (SGD) with a batch size of 32. The training process runs for up to 100 epochs, with the option to terminate prematurely if the validation accuracy does not exhibit improvement over the most recent 10 epochs.

### 2.4. Datasets Used for Benchmarking

We prepared authentic scRNA-seq data from six cell lines along with corresponding cell-specific ChIP-seq data for benchmarking purposes (Table S1) to compare DeepIMAGER with other existing GRN reconstruction methods. These six cell lines encompass human embryonic stem cells (hESC) [33], 5G6GR mouse embryonic stem cells (mESC(2)) [34], two mouse hematopoietic stem cell lines [35] representing granulocyte-macrophage lineage (mHSC(GM)), and lymphoid lineage (mHSC(L)), bone marrow-derived macrophages (BMM) and dendritic cells [36]. ChIP-seq has been widely acknowledged as a gold standard technique for studying cell-specific protein–DNA interactions [37]. The corresponding TF-gene pairs associated with these cell types, as identified by ChIP-seq data, were collected from the gene transcription regulation database (GTRD) [38]. The scRNA-seq and ChIP-Seq datasets of bone marrow-derived macrophages and dendritic cells have undergone preprocessing using CNNC [21]. Similarly, the scRNA-seq expression and ChIP-seq datasets of hESC, mESC(2), mHSC(GM), and mHSC(L) have been preprocessed using an evaluation framework called BEELINE [17].

### 2.5. Comparing with Ten Other Methods

To benchmark DeepIMAGER, we considered five unsupervised GRN inference methods (GENIE3 v3.19 [7], PIDC v1.0 [8], SCODE v1.0 [9], PPCOR v1.1 [10], and SINCERITIES v2.0 [11]) that have consistently demonstrated strong performance in two benchmark comparisons [16,17]. Additionally, we included SCENIC+ v1.0a1 [13] and four newly proposed supervised deep learning methods (CNNC v1.0 [21], DeepDRIM v1.0 [22], dynDeepDRIM v1.0 [23], and GENELink v1.0 [24]). The execution of the five unsupervised methods was facilitated using the interfaces provided by BEELINE [17]. The five supervised deep learning methods, including DeepIMAGER, are all designed for cell-specific GRN inference. The new version of SCENIC, named SCENIC+ [13], can integrate scATAC-seq, scRNA-seq, and TF-binding motifs to predict GRNs. Due to the lack of scATAC-seq data corresponding to the benchmark data, we directly used scRNA-seq data and the precomputed cisTarget TF-binding motif databases of human and mouse. CNNC, DeepDRIM, dynDeepDRIM, and DeepIMAGER share the same data preprocessing programs, and all these methods were assessed using their default parameter settings. A technical summary of these methods can be found in Table S2.

To enhance data quality and expedite the training process, our initial step involved the removal of cells and genes with insufficient information, following the methodology outlined in SCENIC [12], for all the GRN inference tools under comparison. For these supervised learning methods, we adopted the 3-fold cross-validation method for model training. To alleviate the computational burden associated with generating a large number of neighbor images, we randomly selected 13 to 18 TFs from the ChIP-seq data as positive cases (Table S1) and balanced this with the random selection of non-target genes as negative cases. To reduce the occurrence of false positives and ensure a fair evaluation of supervised and unsupervised methods using the same TF-gene pairs, we specifically considered the TF-gene pairs formed by TFs and the top 500 high-variable genes (HVGs). Unsupervised methods were assessed on training sets, while supervised methods were evaluated on test sets. Each method’s default parameters were used for GRN inference unless specified. Detailed commands used for this research can be found at https://github.com/shaoqiangzhang/DeepIMAGER/tree/main/other_methods (accessed on 1 June 2024).

### 2.6. Evaluation Metrics

To ensure a more effective evaluation of the model’s quality and to prevent overfitting or underfitting [39], we employed a 3-fold cross-validation during the model training process. Specifically, a dataset was evenly divided into three subsets, with each subset taking turns as the test set while the remaining two subsets were merged to form the training set. This process was repeated three times, resulting in three models, each associated with a distinct test accuracy value. The final accuracy was computed as the average of these three accuracy values.

In addition to accuracy, we also generated receiver operating characteristic (ROC) curves and calculated the area under the ROC curve (AUROC) [40]. AUROC is considered a superior metric for accuracy, especially for classification problems involving imbalanced datasets, as it assesses the model’s effectiveness in classifying positive samples relative to negative samples at a fixed threshold [41]. To evaluate the robustness of each model, we executed each model ten times on each dataset and represented the corresponding AUROC scores using boxplots.

### 2.7. Real Datasets Used for Inferring GRNs in Cancer Cells

To demonstrate its application in real data analysis, we applied DeepIMAGER to the scRNA-seq datasets for MM patient samples. We downloaded the scRNA-seq data with the GEO accession number of GSE124310, which was sampled from 7 MM patients, to predict the GRN of dendritic cells (DC) in MM [42]. The annotation file of cell types was downloaded from the reference’s extended data [42]. The scRNA-seq data preprocessing is the same as in Section 2.2. We inputted the preprocessed scRNA-seq data of the 168 cells to the trained model of dendritic cells to predict dendritic-cell-specific GRN in MM. To detect potential GRNs associated with MM pathology, we examined the top 100 DEGs in MM patients using SCANPY and selected three TFs, *MITF*, *RORC*, and *FOXD2*, for GRN inference. The GRN networks of MITF, RORC, and FOXD2 were plotted using Cytoscape [43].

## 3. Results

### 3.1. DeepIMAGER Can Effectively Infer GRNs

**Figure 2:**
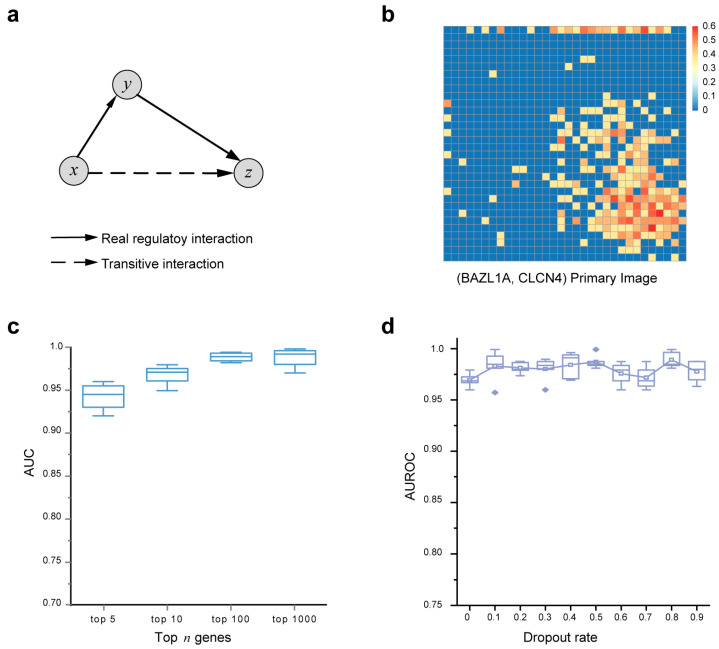
The effect of neighbor images on GRN reconstruction in DeepIMAGER. (**a**) An example of transitive interaction. Gene x and gene z correlate with each other through an intermediate gene y but may be false positives. (**b**) Demo examples of the primary image for (BAZL1A, CLCN4). Their self-images and neighbor images are presented in Figure S2. (**c**) AUROC scores for different numbers of neighbor images on the BMM dataset. (**d**) The AUROC scores with different dropout rates are used in dropout layers. The points outside the boxes are outliers.

Although many statistical and deep learning-based methods have been developed to infer GRNs from both bulk and single-cell RNA-seq data, they often suffer from low precision [16,17]. This challenge is particularly apparent in many correlation-based methods, such as SCENIC [12] and SCODE [9], which rely heavily on pairwise correlation measures like Pearson correlation, Spearman correlation, or mutual information to identify co-expression patterns among genes. As shown in Figure 2a, the expression levels between gene x and gene z may exhibit a strong correlation due to the regulatory interactions existing between gene x and gene y, as well as between gene y and gene z. Consequently, the interaction between gene x and gene z becomes transitive, even though there may be no direct regulatory relationship between gene x and gene z in the real GRN. This results in unpredictable noises and false positive discoveries [44]. To effectively utilize gene-gene correlation information while filtering false positives, we introduce an image-transformation-based strategy that represents the correlations of a gene with other genes as correlation images. The working hypothesis is that using relationships among multiple genes will improve the precision of inferring true regulatory relationships between genes compared to using single-paired gene correlations. Specifically, we represent the co-expressed profile of a target gene pair (x,y) using two primary images for (x,x) and (y,y). We also construct neighbor images, consisting of n images derived from gene x and n genes that have the highest covariance with gene x, as well as n images generated from gene y and n genes that have the highest covariance with gene y (Figure 2b and Figure S2). This approach yields a total of 2n+2 neighbor images generated from a single primary image, effectively retaining potential regulatory information. Subsequently, we leverage the high performance of the ResNet network framework [31] to identify target TF-gene pairs and their closely associated neighboring genes, reducing the occurrence of false positives in GRN inference.

Establishing the deep learning framework involves a crucial step in the proper selection of models to achieve optimal performance. We hypothesize that the number n of neighborhood contexts, or equivalently, the number 2n+2 of neighbor images, plays a pivotal role in influencing the performance of GRN inference. To investigate this, we selected the BMM dataset, which boasts the largest number of cells among the six benchmark datasets, to assess the model performance with varying numbers of input neighbor images for each primary image. As depicted in Figure 2c, increasing the number of invoked neighbor images enhances DeepIMAGER performance. In other words, DeepIMAGER’s performance benefits from involving more neighboring genes for each target TF-gene pair; however, it is noteworthy that DeepIMAGER’s performance does not exhibit significant improvement after involving more than 100 genes, indicating that the top 100 neighboring genes could be an ideal practical parameter for not only achieving good performance but also maintaining low runtimes (Figure 2c).

To mitigate the risk of overfitting or underfitting during model training, we introduced a dropout layer following the average pooling layer and each fully connected layer, in addition to implementing the 3-fold cross-validation method for overfitting reduction. We tested DeepIMAGER with varying dropout rates and observed that it consistently maintains stable performance across diverse dropout configurations (Figure 2d). On all testing datasets, the AUROC scores are greater than 0.95. These multiple dropout-based validations also show that DeepIMAGER is not prone to excessive overfitting because they obtain similar performance across different testing datasets.

### 3.2. DeepIMAGER Has Superior Performance than Ten Existing Methods

**Figure 3:**
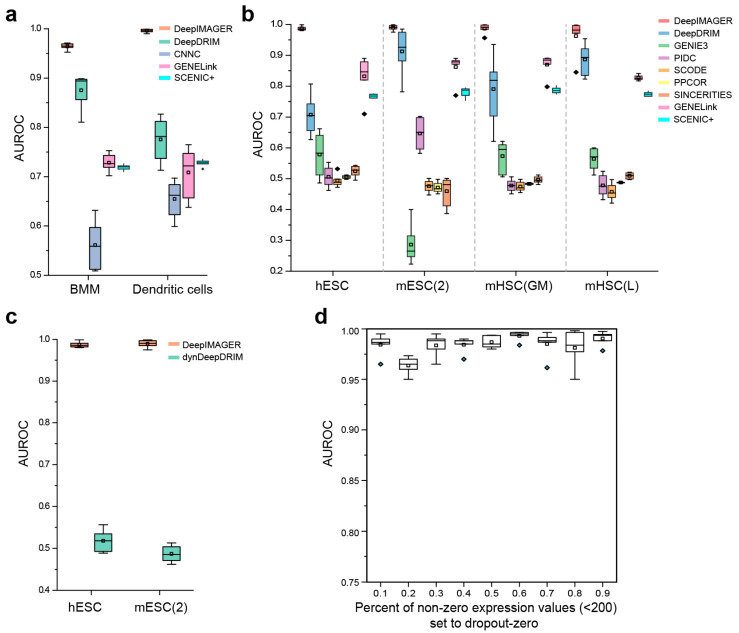
Performance comparison of ten methods. (**a**) The AUROC scores of five supervised GRN inference methods on two scRNA-seq datasets without pseudo-time-ordered information. (**b**) The AUROC scores of five unsupervised and three supervised GRN inference methods on four pseudotime-ordered scRNA-seq datasets. (**c**) The AUROC scores of dynDeepDRIM on two pseudo time-ordered scRNA-seq datasets. (**d**) The AUROC scores of DeepIMAGER on data synthesized by BMM with different percentages of dropout zeros. The points outside the boxes are outliers.

We conducted a comprehensive comparison of DeepIMAGER with a set of well-established unsupervised GRN reconstruction methods, including GENIE3 [7], PIDC [8], SCODE [9], PPCOR [10] and SINCERITIES [11], along with recently proposed supervised cell-specific GRN reconstruction methods like CNNC [21], DeepDRIM [22], dynDeepDRIM [23], SCENIC+ [13] and GENELink [24]. Initially, we evaluated DeepIMAGER against three supervised deep learning methods (excluding dynDeepDRIM, designed for pseudotime-ordered scRNA-seq data) using two datasets without pseudotime information, namely BMM and dendritic cells. As illustrated in Figure 3a, DeepIMAGER achieved significantly higher median AUROC scores of 0.97 and 0.99, with small variances for BMM and dendritic cells, respectively. DeepIMAGER outperforms the other four supervised methods (CNNC, DeepDRIM, SCENIC+, and GENELink) by a substantial margin.

Subsequently, we extended our evaluation to include four pseudotime-ordered scRNA-seq and ChIP-seq datasets, namely hESC, mESC(2), mHSC(GM), and mHSC(L), which had been preprocessed and subjected to an ensemble of five unsupervised methods through the BEELINE framework. Additionally, we ran DeepIMAGER, DeepDRIM, and GENELink on these four preprocessed datasets. As demonstrated in Figure 3b, DeepIMAGER consistently achieved median AUROC scores ranging from 0.97 to 0.99 across the four datasets, surpassing all other supervised and unsupervised methods by a significant margin. DeepDRIM emerged as the second-best performer, with median AUROC scores ranging from 0.71 to 0.92 across the six datasets. For dynDeepDRIM, a specialized version of DeepDRIM designed for pseudotime-ordered scRNA-seq data, we limited its evaluation to two pseudotime-ordered datasets, namely hESC and mESC(2), due to its considerable computational demands. As depicted in Figure 3b,c, both DeepIMAGER and DeepDRIM significantly outperformed dynDeepDRIM in terms of AUROC scores. Notably, we observed that the variances of DeepIMAGER AUROC scores are consistent across all six datasets, exhibiting remarkable robustness compared with the other supervised methods. It is worth mentioning that multiple studies have consistently shown that supervised methods tend to outperform unsupervised tools on both simulated and real scRNA-seq data [18,22,24,45], which is supported by our experiments (Figure 3b). Overall, these extensive comparative analyses demonstrate DeepIMAGER’s superiority over other methods, particularly in the context of cell-specific GRN inference.

### 3.3. DeepIMAGER Is Robust against High Dropout Noise in scRNA-seq Data

Due to experimental limitations, scRNA-seq data commonly contains dropout-zero events, wherein the expression of a gene appears to be zero in a single cell despite its known expression in bulk samples or other cells. To assess the impact of dropout zeros on DeepIMAGER’s performance, we conducted experiments using the BMM dataset, which encompasses 20,463 genes and 6283 cells. In this analysis, we deliberately replaced certain non-zero values in the gene expression matrix with dropout zeros. Given that dropout-zero events tend to occur more frequently for genes with low expression levels, we introduced dropout noise by setting different percentages (ranging from 10% to 90% in increments of 10%) of non-zero elements with expression counts less than 200 to zero. For each percentage level, we generated 10 random replicates and calculated their respective AUROC scores. The results show that, despite the introduction of varying percentages of dropout-zeros, all AUROC scores consistently remained higher than 0.95 (Figure 3d). This observation suggests that the retention or removal of low-expression values has minimal impact on DeepIMAGER’s performance. Further, it highlights that the replacement of low expression counts with dropout zeros did not disrupt the fundamental characteristics of a 32 × 32-pixel image, which serves as a representation of the joint expression of two genes.

### 3.4. Technical Comparison of DeepIMAGER and DeepDRIM

**Figure 4:**
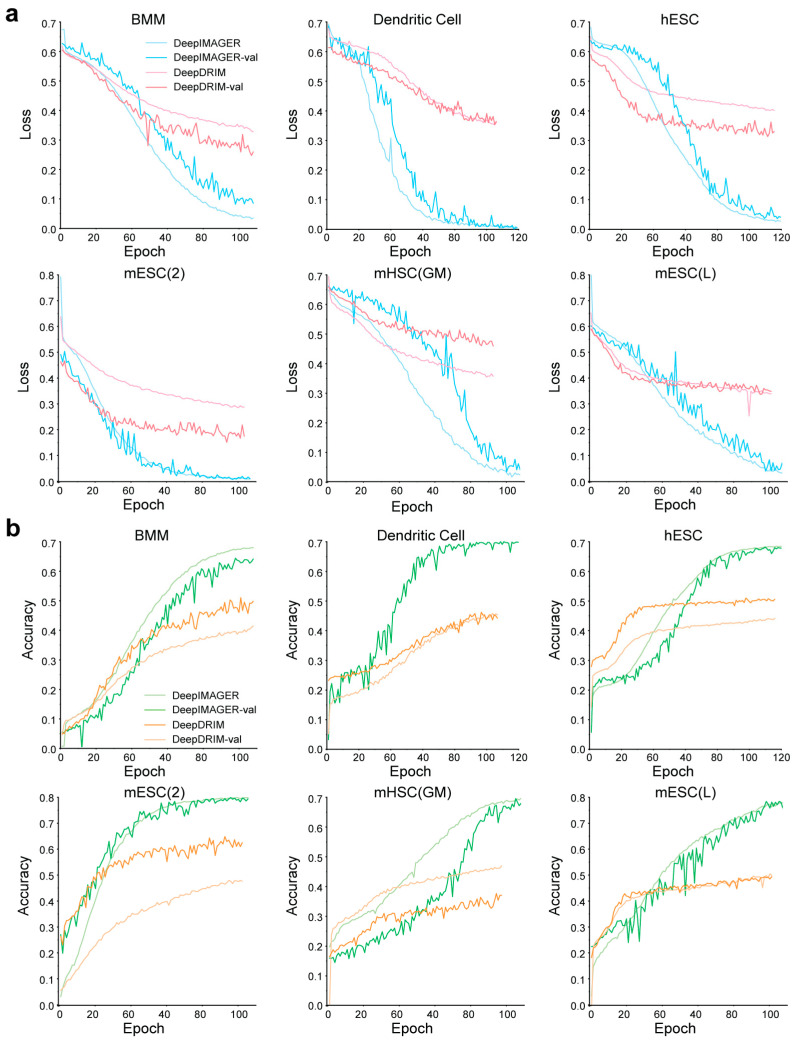
Technical comparison of DeepIMAGER and DeepDRIM. (**a**) The loss and validation loss (val_loss) curves of DeepIMAGER and DeepDRIM vary with the increasing number of epochs on six scRNA-seq datasets. (**b**) The accuracy and validation accuracy (val_accuracy) curves of DeepIMAGER and DeepDRIM vary with an increasing number of epochs on six scRNA-seq datasets.

Among the compared supervised deep learning methods, we noticed that although DeepDRIM’s performance is lower than our method, it consistently ranks second among all methods. Since both DeepDRIM and DeepIMAGER address binary classification problems employing the same loss function, namely binary cross-entropy loss, we assessed their technical differences during training across all six experimental datasets. As depicted in Figure 4a, for each of the six datasets, there is a downward trend in both the training loss values (illustrated by the loss curve) and the validation loss values (indicated by the val_loss curve) as the number of epochs increases for both DeepIMAGER and DeepDRIM. However, the curves for DeepIMAGER exhibit steeper declines and approach closer to zero. Notably, the loss and val_loss curves for DeepDRIM become relatively flat after approximately 40 epochs, especially evident in the fact that the val_loss values for DeepDRIM are smaller than the corresponding loss values in five out of the six datasets, suggesting overfitting in DeepDRIM. Conversely, DeepIMAGER demonstrates a decreasing trend in both val_loss and loss curves before reaching the maximum 100 epochs. Moreover, the val_loss curves for DeepIMAGER are generally higher than the corresponding loss curves in five of the six datasets, making overfitting unlikely. The only case where the two curves for DeepIMAGER are close is mESC(2).

For the six datasets, we also plotted the curves representing training accuracy (referred to as accuracy curves) and validation accuracy (referred to as val_accuracy curves) with increasing epoch counts (Figure 4b). These curves demonstrate that both accuracy and validation accuracy increase with a higher number of epochs. However, after 60 epochs, DeepIMAGER’s accuracy and validation accuracy surpass those of DeepDRIM, even nearing 1 at the 100th epoch in most datasets. Figure 4b also reveals that DeepDRIM might exhibit overfitting in five datasets, except for mESC(GM), as indicated by val_accuracy curves consistently exceeding the corresponding accuracy curves. Conversely, DeepIMAGER is less likely to experience overfitting, given that its val_accuracy curve generally lags behind the corresponding accuracy curve in each dataset.

### 3.5. DeepIMAGER Detected Potential GRNs for Three TFs in Dendritic Cells of the Multiple Myeloma Microenvironment

To demonstrate DeepIMAGER’s ability to uncover novel biological interactions, we applied it to MM scRNA-seq datasets to detect potential GRNs. Based on the authors’ annotations, we selected 168 dendritic cells (DCs) to predict GRNs and specifically focused on TFs that are differentially expressed in MM patients. DCs play a crucial role in the immune landscape of MM [46,47], involved in antigen presentation, immune response modulation, and interactions with the tumor microenvironment. Therapeutic strategies targeting to enhance their function or overcome their immunosuppressive effects hold promise for improving MM treatment outcomes [47,48]. To detect potential GRNs associated with MM pathology, we examined the genes that show significant expression changes (DEGs) in MM patients compared to healthy controls. Among the top 100 DEGs, we selected three TFs, *MITF*, *RORC*, and *FOXD2*, to infer their GRNs.

**Figure 5:**
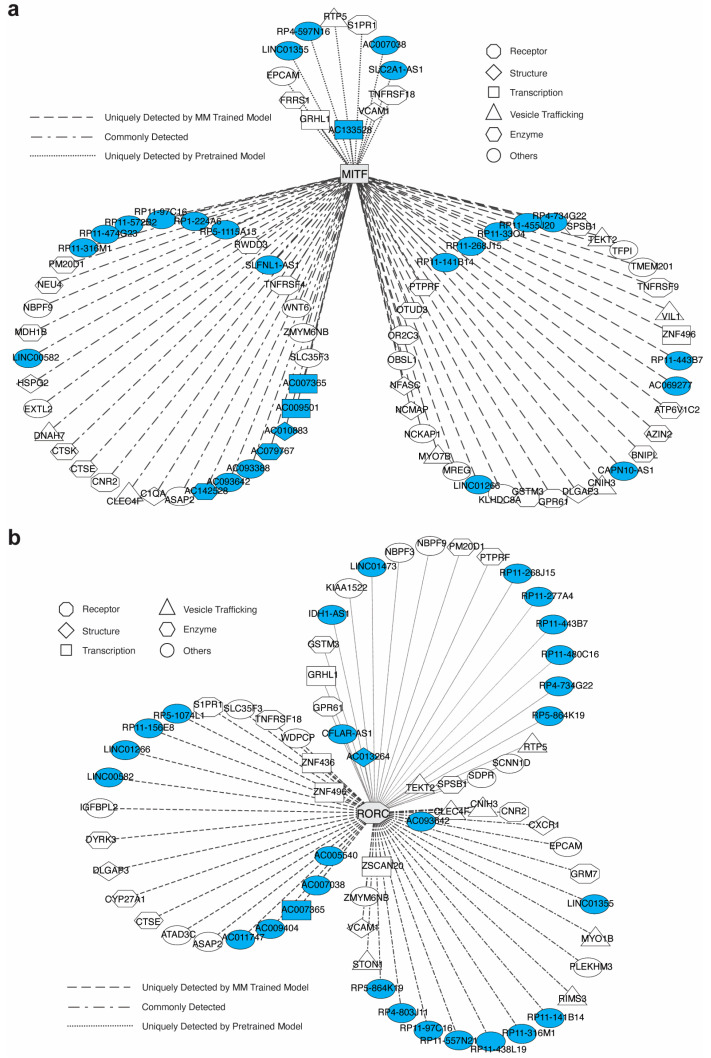
Predicted GRNs for *MITF* and *RORC*. (**a**) The GRNs for *MITF*. (**b**) The GRNs for *RORC*. The non-coding genes are marked in blue.

First, we examined the MITF gene (Microphthalmia-associated transcription factor), which plays a crucial role in regulating gene expression and has been associated with cancer development, such as melanoma [49,50]. However, it has been poorly studied in MM. We found a total of 78 predicted regulations in the *MITF* GRN, including 33 regulations only detected by the MM-trained model, 12 regulations only detected by the pre-trained model, and 33 common regulations (Figure 5a). These targeted genes are mainly classified into functional categories such as receptor, transcription-related, enzyme activity, structure-related, vesicle trafficking, and others. Several TNF receptor superfamily members (i.e., *TNFRSF18*, *TNFRSF4*, *TNFRSF9*) were previously identified in the RANKL/RANK/OPG pathway, which is one of the key pathways through which *MITF* influences MM pathology [51]. Interestingly, we found that many *MITF* targets are long intergenic non-coding RNAs (LINCs), antisense RNAs, and annotated chromosomal transcriptional regions, such as *RP11-572B2*, *RP11-97C16*, *AC079767*, *AC007365*, *LINC00582*, *SLFNL1-AS1*, and others.

Second, we examined the *RORC* gene (Retinoic Acid Receptor-Related Orphan Receptor Gamma), which encodes a TF in the nuclear receptor superfamily. In MM pathology, the involvement of *RORC* is primarily related to its role in regulating the tumor microenvironment and immune responses [52]. We identified a total of 67 predicted regulations in the *RORC* GRN, including 22 regulations only detected by the MM-trained model, 23 regulations only detected by the pre-trained model, and 22 regulations commonly detected (Figure 5b). In the literature review, we found that many of these predictions have been linked to MM pathology. For example, the *WNT6* gene belongs to the WNT gene family, which encodes secreted signaling proteins that play crucial roles in various developmental and physiological processes in MM [53].

Third, we predicted GRNs for *FOXD2*, which encodes a member of the forkhead box (FOX) family of TFs and plays an important role in regulating gene expression and controlling various cellular processes, including cell proliferation, differentiation, and survival [54,55]. We identified a total of 67 predicted regulations in the *FOXD2* GRN, including 47 regulations only detected by the MM-trained model and 33 regulations commonly detected (Figure S3). Meanwhile, the predicted targets of *RORC* and *FOXD2* are also associated with diverse functional categories, indicating their broad impacts on regulations.

Interestingly, we found that many LINC RNAs, antisense RNAs, and novel chromosomal transcriptional regions are enriched in the three GRNs of *MITF*, *RORC*, and *FOXD2* (Figure 5a,b and Figure S3). Specifically, there are a total of 29 non-coding genes in the *MITF* GRN, 28 in the *RORC* GRN, and 25 in the *FOXD2* GRN, many of which are associated with MM pathology. For example, the *LINC00582* gene, detected in both *MITF* and *RORC* GRNs, has been reported in previous non-coding RNA transcriptome studies in MM [56]. Additionally, *IDH1-AS1*, one of the regulatory targets of *RORC*, has been well investigated in tumor metabolism and immunity [57,58,59]. Given that LINC RNAs and antisense RNAs are increasingly recognized for their involvement in diverse cancers [60,61,62] and MM [63,64,65,66], the identification of these non-coding elements provides novel candidates and valuable insights into potential mechanisms underlying disease pathogenesis. Further research is needed to fully investigate the roles of these non-coding RNAs in MM development and progression, as well as to explore their potential as therapeutic targets or biomarkers.

## 4. Discussion

We introduced DeepIMAGER, a novel method for inferring GRNs from scRNA-seq data. Our approach leverages two key strategies: an image-transformation-based procedure and the ResNet network framework. Through extensive validation on six different cell lines, DeepIMAGER consistently outperformed ten popular methods. Notably, DeepIMAGER achieved exceptionally high AUROC scores, reaching up to 99% for inferring GRNs. This remarkable performance indicates that our method effectively mitigates the issue of false positives that are often encountered by other GRN inference methods. Furthermore, we applied DeepIMAGER to MM scRNA-seq data and obtained GRNs associated with TFs of *MITF*, *RORC,* and *FOXD2*. As many regulations in these GRNs have been previously reported, our predictions may be highly reliable, represent novel discoveries, and provide a potential therapeutic strategy for MM treatment. For example, modulating *RORC* activity or inhibiting downstream effectors could potentially disrupt the tumor-promoting effects of the tumor microenvironment and enhance the anti-tumor immune response [52].

One of the key technical challenges in employing deep learning strategies lies in the design of network structures, particularly the determination of the number of layers, which directly impacts model performance. In DeepIMAGER, we adopted a ResNet50 architecture, which demonstrated significantly better performance compared to ResNet18. ResNet18 consists of a network depth of 18 layers, with each basic block composed of two 3 × 3 convolutional layers. In contrast, ResNet50 includes an internal residual block with two 1 × 1 convolutional kernels and a 3 × 3 convolutional kernel [31] (Figure S4a). When we replaced ResNet50 with ResNet18, we observed a dramatic reduction in AUROC scores for the BMM and mESC(2) datasets (Figure S4b). This emphasizes the critical importance of carefully designing the network structure, specifically the depth of layers when practically applying deep learning strategies.

Like other supervised methods, DeepIMAGER is a model trained on scRNA-seq data from a specific cell type, making it a cell-type-specific model. To assess the model’s potential for transfer learning, we trained DeepIMAGER on the mESC(2) dataset and then applied it to mHSC(L) and vice versa. As illustrated in Figure S4c,d in the Supplementary File, applying DeepIMAGER trained on one cell type to another cell type yields considerably worse results compared to models trained and tested within the same cell type. This indicates that DeepIMAGER may not be suitable for transfer learning applications across distinct cell types.

Future research in GRN inference should target challenges associated with integrating diverse data types and conducting comparative analyses. First, the integration of diverse data types holds great promise for advancing GRN inference. Beyond the TF ChIP-seq data used in this study, additional omics data such as scATAC-seq [67], scHi-C [68], and single-cell DNA methylation [69] can be harnessed for GRN inference. This expanded data integration can not only enhance performance but also deepen our understanding of gene regulation in both health and disease contexts. Second, conducting comparative analyses of GRNs under various biological conditions represents another promising avenue for future research [70,71]. This approach can yield insight into condition-specific regulatory mechanisms and facilitate the identification of key regulators driving phenotype variation. For instance, a comparative analysis of GRNs in disease states versus healthy states holds the potential to unveil dysregulated pathways and identify therapeutic targets [72]. Similarly, exploring GRNs among different cell types within the same tissue or organ can unveil cell-specific regulatory interactions [73]. Fostering collaborative efforts between computational biologists, experimental biologists, and clinicians will be essential for translating GRN research findings into clinical applications and personalized medicine. This interdisciplinary approach will facilitate the practical utilization of GRN insights in disease diagnosis, targeted therapy development, and precision medicine advancement initiatives.

## Data Availability

The scRNA-seq and ChIP-seq datasets of hESC, mESC(2), mHSC(GM), and mHSC(L) are available at https://doi.org/10.5281/zenodo.3378975. scRNA-seq and ChIP-Seq data of bone marrow-derived macrophages and dendritic cells are available at https://github.com/xiaoyeye/CNNC (accessed on 10 November 2023). The scRNA-seq dataset of Multiple Myeloma is available in Gene Expression Omnibus (GEO) with the accession number GSE124310. The Python source code and usage instructions for DeepIMAGER are available at https://github.com/shaoqiangzhang/DeepIMAGER (accessed on 1 February 2024).

## Supplementary Materials

The following supporting information can be downloaded at: https://www.mdpi.com/article/10.3390/biom14070766/s1, Tables S1 and S2 and Figures S1–S4. Table S1: scRNA-seq datasets from six cell lines used in benchmarking. Table S2: Technical summary of ten methods. Figure S1: Network structure of DeepIMAGER. Figure S2: Demo examples of self-images and neighbor images for BAZL1A and CLCN4. Figure S3: Predicted GRN of FOXD2. Figure S4: Evaluations of model selection and cell-specific training.
