# Supplementary material for: DeepIMAGER: Deeply Analyzing Gene Regulatory Networks from scRNA-seq Data
Source: Biomolecules. 2024 Jun 27;14(7):766. doi: 10.3390/biom14070766 (PMC11274664; doi:10.3390/biom14070766)
Supplement: Supplementary file 1 [file biomolecules-14-00766-s001.zip › biomolecules-3029642-supplementary.pdf]

biomolecules

Supplementary Data

Table S1. scRNA-seq datasets from six cell lines used in benchmarking.

Datasets	PMID	Number of cells	Number of genes	Number of TFs	Pseudo-time
BMM	30425249	6283	20463	13	N
Dendritic cells	30425249	4126	20463	16	N
hESC	27534536	758	17735	18	Y
mESC(2)	29434199	421	18385	18	Y
mHSC(GM)	27365425	889	4762	18	Y
mHSC(L)	27365425	847	4762	18	Y

Table S2. Technical summary of ten methods. S: Supervised, US: Unsupervised. ODE: Ordinary Differential Equation.

Tool	PMID	S or US	Technical Features	Language	Year	URLs
GENIE3	20927193	US	random tree	R	2010	CRAN
PIDC	28957658	US	co-expression	Julia	2017	https://github.com/Murali-group/Beeline/blob/master/Algorithms/PIDC/
SCODE	28379368	US	ODE	R	2017	CRAN
PPCOR	26688802	US	semi-partial correlation calculation	R	2015	CRAN
SINCERITIES	28968704	US	Pseudo temporal gene correlation	Matlab/R	2018	https://github.com/CABSEL/SINCERITIES
SCENIC+	37443338	S	combines single-cell chromatin accessibility and gene expression data with motif discovery	Python	2023	https://github.com/aertslab/scenicplus
CNNC	31822622	S	deep learning	Python	2019	https://github.com/xiaoyeye/CNNC
DeepDRIM	34424948	S	deep learning	Python	2021	https://github.com/jiaxchen2-c/DeepDRIM
dynDeepDRIM	36168811	S	deep learning	Python	2022	https://github.com/ericcombiolab/dynDeepDRIM
GENELink	35961023	S	deep learning	Python	2022	https://github.com/zpliulab/GENELink

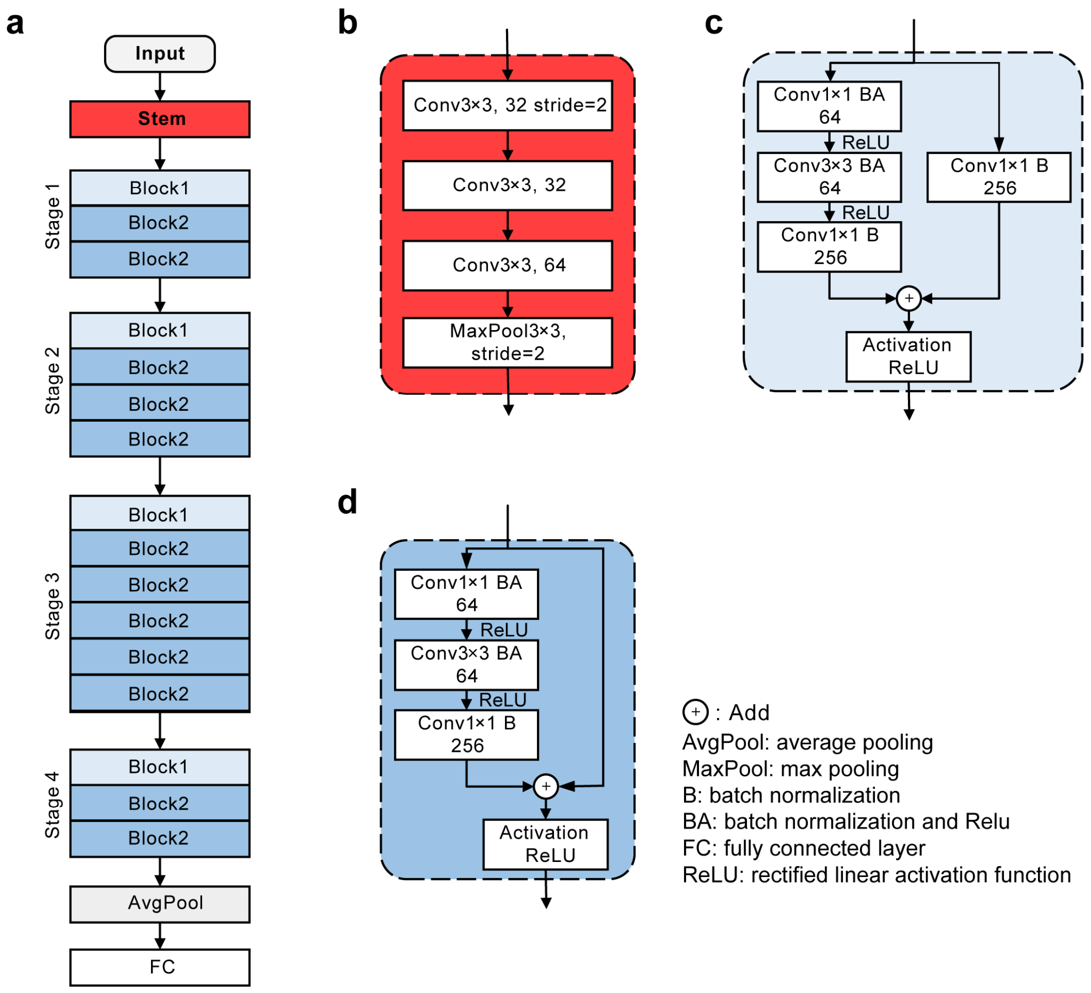

Figure S1. Network structure of DeepIMAGER. (**a**) The framework of networks X and Y revised from ResNet50. (**b**) The structure of Stem. (**c**) the structure of Block1. (**d**) The structure of Block2.

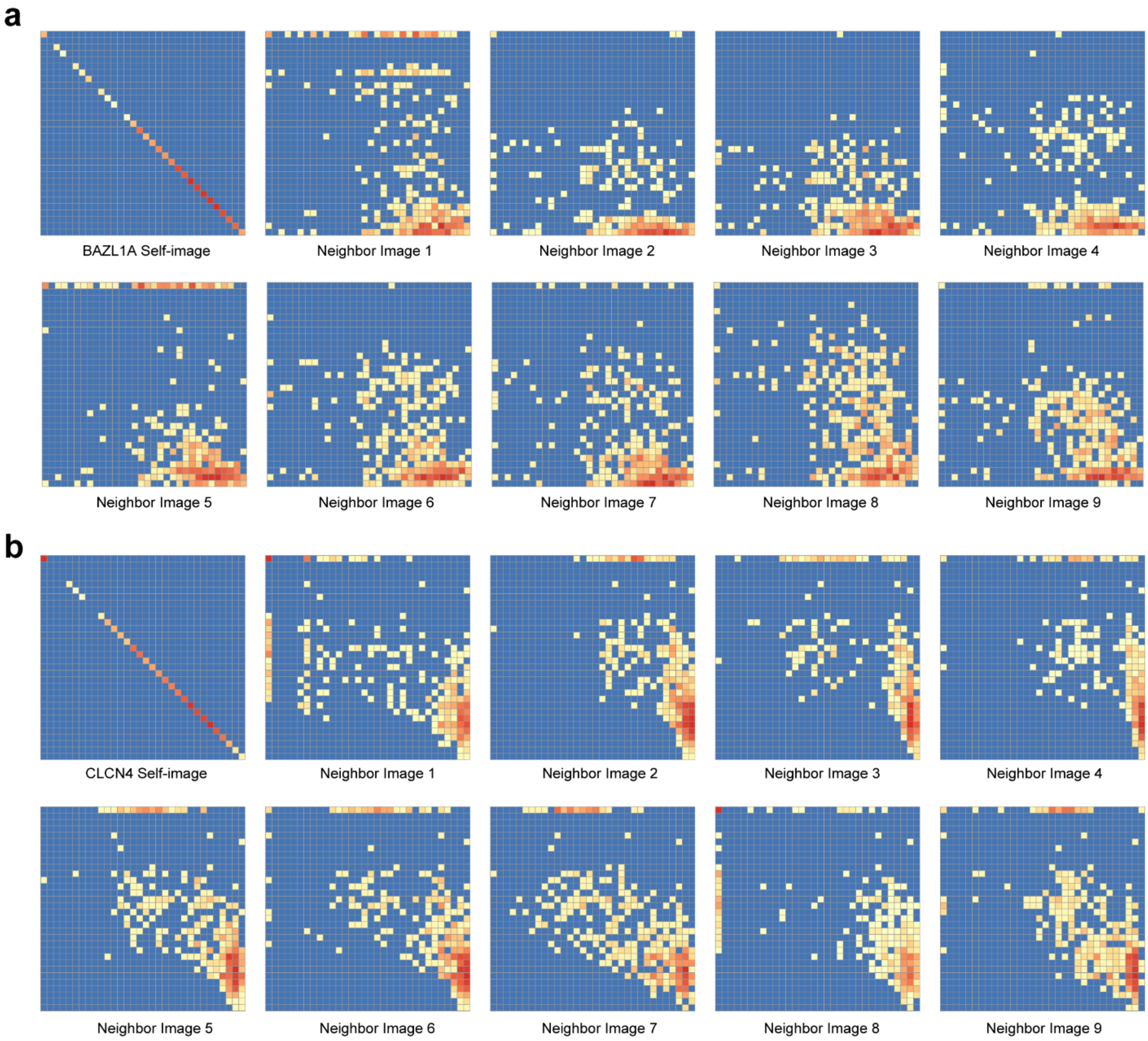

Figure S2. Demo examples of self-images and neighbor images for BAZL1A (**a**) and CLCN4 (**b**).

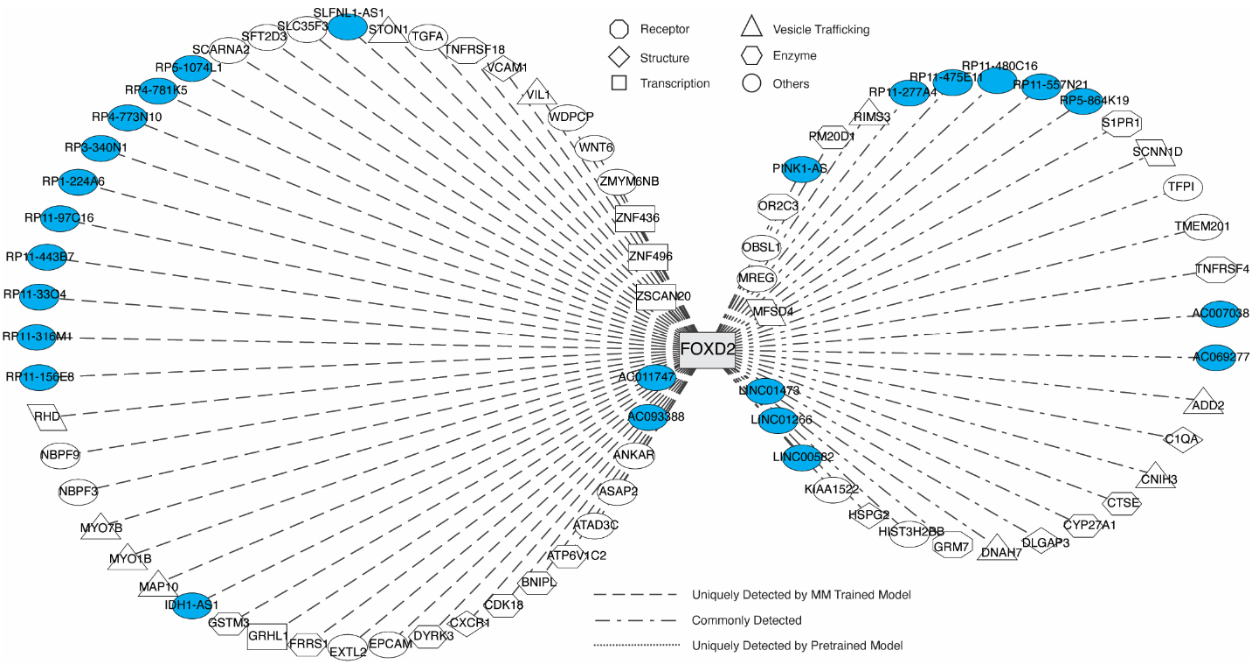

Figure S3. Predicted GRN of FOXD2. The non-coding genes marked in blue.

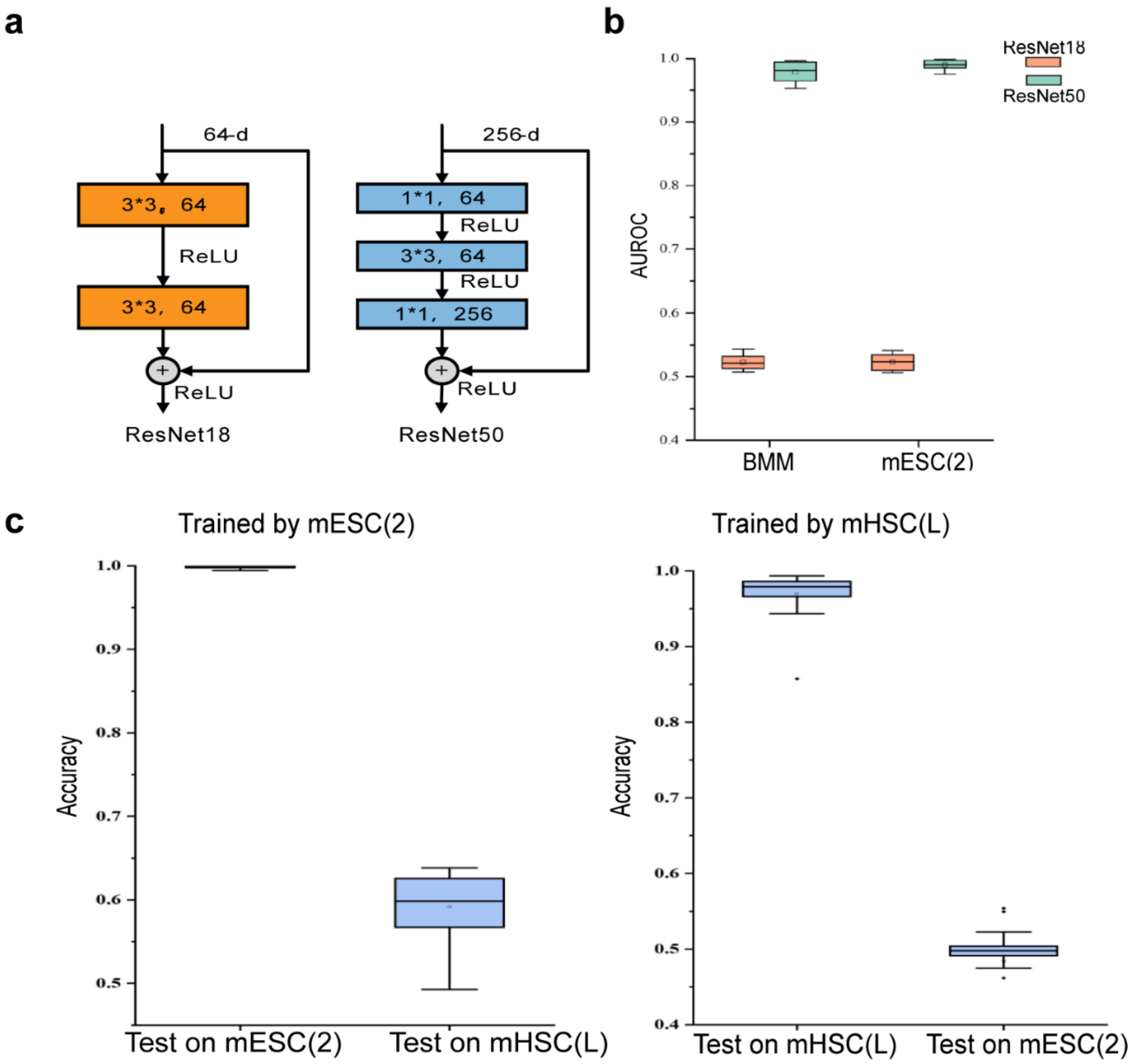

Figure S4. Evaluations of model selection and cell-specific training. (**a**) Differences in the internal construction of the residual blocks of ResNet18 and ResNet50. (**b**) Comparison of experimental results between ResNet18 and ResNet50 on two datasets. (**c**) The accuracy of DeepIMAGER that trained and tested on the same and different cell types.
